# New‐onset atrial fibrillation and outcomes following isolated coronary artery bypass surgery: A systematic review and meta‐analysis

**DOI:** 10.1002/clc.23414

**Published:** 2020-07-21

**Authors:** Matthew Kerwin, Jonathan Saado, Jonathan Pan, Gorav Ailawadi, Sula Mazimba, Michael Salerno, Nishaki Mehta

**Affiliations:** ^1^ Division of Internal Medicine University of Virginia Charlottesville Virginia USA; ^2^ Division of Cardiovascular Surgery University of Virginia Charlottesville Virginia USA; ^3^ Division of Cardiovascular Medicine University of Virginia Charlottesville Virginia USA; ^4^ Division of Cardiovascular Medicine William Beaumont Oakland University Royal Oak MI USA

**Keywords:** atrial fibrillation, coronary artery bypass surgery, postoperative atrial fibrillation, stroke

## Abstract

Prior meta‐analyses have shown that new‐onset atrial fibrillation (NOAF) occurs in up to 40% of patients following cardiac surgery and is associated with substantial major adverse cardiovascular events. The stroke and mortality implications of NOAF in isolated CABG without concomitant valve surgery is not known. We thought that NOAF would be associated with increased risk of stroke and mortality, even in patients undergoing isolated CABG. A blinded review of studies from MEDLINE, CENTRAL, and Web of Science was done by two independent investigators. Stroke, 30‐day/hospital mortality, long‐term cardiovascular mortality, and long‐term (>1 year) all‐cause mortality were analyzed. We used Review Manager Version 5.3 to perform pooled analysis of outcomes. Of 4461 studies identified, 19 studies (n = 129 628) met inclusion criteria. NOAF incidence ranged from 15% to 36%. NOAF was associated with increased risk of stroke (unadjusted OR 2.15 [1.82, 2.53] [*P* < .00001]; adjusted OR 1.88 [1.02, 3.46] [*P* = .04]). NOAF was associated with increased 30‐day/hospital mortality (OR 2.35 [1.67, 3.32] [*P* < .00001]) and long‐term cardiovascular mortality (OR 2.04 [1.35, 3.09] [*P* = .0007]) NOAF was associated with increased long‐term all‐cause mortality (unadjusted OR 1.79 [1.63, 1.96] [*P* < .00001]; adjusted OR 1.58 [1.24, 2.00] [*P* = .0002]). We found that the incidence of NOAF following isolated CABG is high and is associated with increased stroke rate and mortality. Early recognition and management of NOAF could improve outcomes.

## 1 INTRODUCTION

Following coronary artery bypass grafting (CABG), patients frequently develop new‐onset atrial fibrillation (NOAF). Of approximately 800 000 patients who undergo CABG each year, over 264 000 with develop NOAF.^1^ The clinical implications of this phenomenon are significant, given that the development of NOAF has been associated with up to a 56% greater risk of long‐term mortality.^2^ NOAF has also been associated with increased risk of stroke. Three prior meta‐analyses have examined outcomes of patients who develop NOAF after CABG in a mixed group of patients undergoing cardiac and thoracic procedures concomitantly.^3^, ^4^, ^5^ However, each of these analyses included patients who had undergone concomitant valvular surgery, which could prognosticate for the development of atrial fibrillation (AF). The incidence of AF has been shown to be higher in those patients undergoing valvular surgery (46%) than in those patients undergoing isolated CABG (29%).^6^ Our goal was to perform a systematic review and meta‐analysis to provide the most current information surrounding outcomes in patients with NOAF undergoing isolated CABG. We excluded patients who underwent concomitant procedures, including valve repair or replacement, aneurysmectomy, or arrythmia surgery. This is the first meta‐analysis to focus on development of NOAF in patients undergoing isolated CABG.

While many studies have shown increased mortality and stroke incidence in patients with NOAF, fewer studies have reported adjusted outcomes. Adjusting for confounding variables is essential given the multiple comorbidities that may affect outcomes in this patient population. We hypothesized that patients with NOAF following isolated CABG would have a higher incidence of mortality and of stroke, even when outcomes have been adjusted.

## 2 METHODS

### 2.1 Eligibility criteria

We included studies that reported our outcomes of interest in patients with NOAF following isolated CABG. Any study that did not specify whether the procedure was an isolated CABG, or that included patients undergoing isolated CABG but did not stratify outcomes by that subgroup, was excluded. We excluded studies in which patients underwent concomitant valve surgery, given that the additional atriotomy sites and annulus manipulation act as independent risk factors for development of atrial arrhythmias. Studies that contained populations of vascular surgery patients were excluded. We excluded studies in which the population did not explicitly exclude those patients with a prior history of AF. We also excluded studies in languages other than English. We excluded abstracts and unpublished studies. We reviewed citations of included articles, which did not yield any new relevant studies.

### 2.2 Data collection and analysis

This review follows the MOOSE guidelines for meta‐analysis reporting.^7^ Approval by an ethics committee was not required. A single author (Matthew Kerwin) abstracted data. Data was verified by a second author (Jonathan Saado). We collected data on author, year, study design, outcomes measured, follow‐up rate, and several other fields (Table S3). We collected absolute event counts. Several studies reported both adjusted and unadjusted data. In these cases, we collected both sets of data. When necessary, we contacted authors to clarify results. We assessed study quality using the Newcastle‐Ottawa scale. The assessment of study quality was done by two blinded investigators (Matthew Kerwin and Jonathan Saado). Any disagreements were resolved by consensus.

Statistical analysis was performed using Review Manager (Rev Man, version 5.3, London, UK). We performed pooled analysis of adjusted and, when available, unadjusted outcomes. Hazard ratios, odds ratios, and relative risk were assumed to be roughly equivalent. We used a random‐effects model, assuming that the analyzed studies were examining different populations. We determined 95% confidence intervals for all estimates. Heterogeneity was assessed using the *I* ^2^ statistic.

## 3 RESULTS

### 3.1 Characteristics of included studies

**FIGURE 1:**
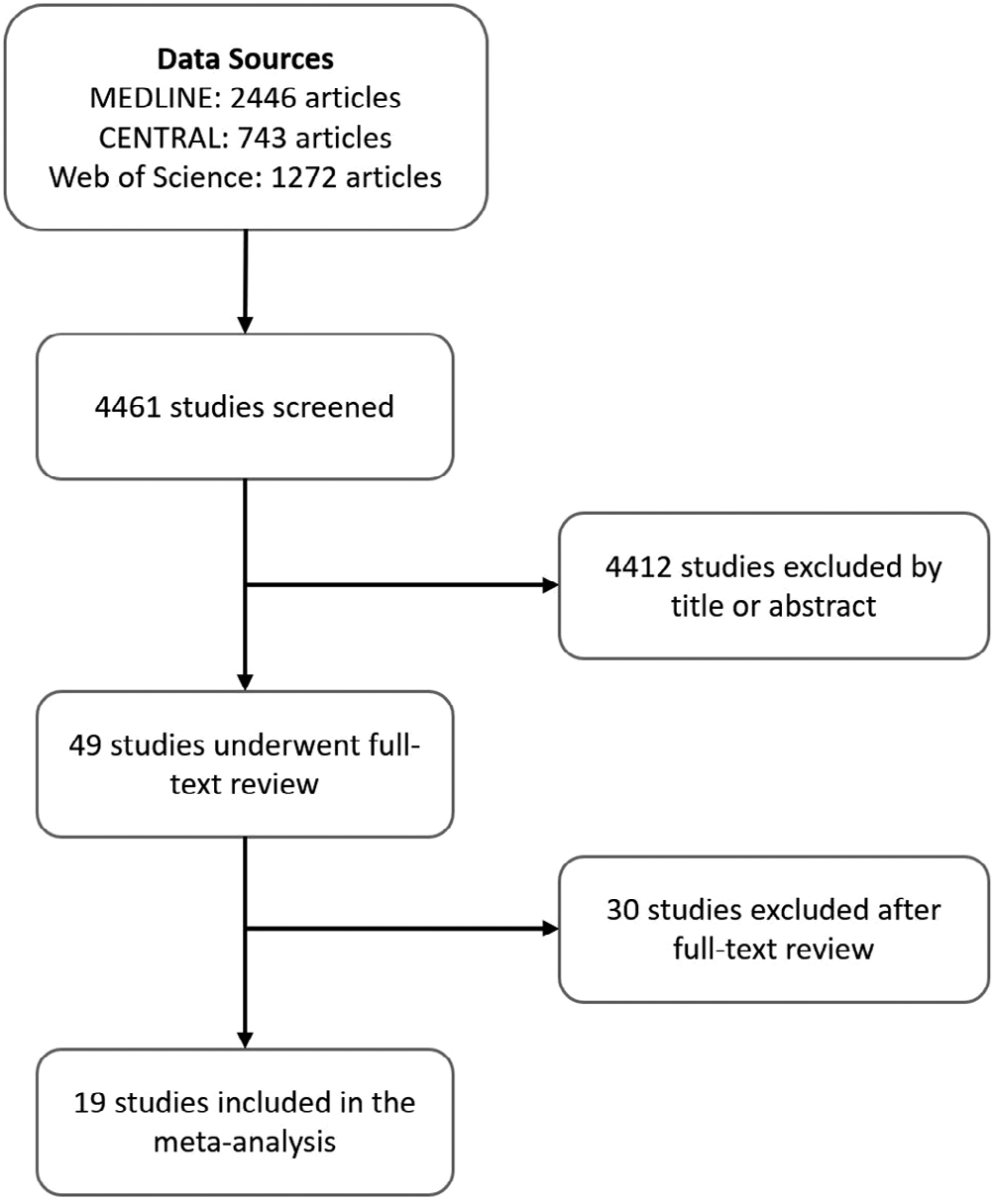
Study selection flowchart

Of the 4461 studies identified, 49 studies were adjudicated as relevant and underwent full‐text review (Figure 1). Of these, 19 studies met the criteria and were included in the meta‐analysis. Any conflicts about inclusion status were resolved by consensus. These studies comprised 129 628 patients. Eleven studies reported on stroke outcomes. Five reported hospital or 30‐day mortality and 16 reported long‐term all‐cause mortality. Thirteen studies reported adjusted variables. The most common variables for which authors adjusted were age, sex, history of myocardial infarction, diabetes, hypertension, and tobacco use. We abstracted additional details for each study. All but one study was retrospective. The majority had been conducted at a single center.

### 3.2 Incidence of NOAF and use of anticoagulation

NOAF incidence ranged from 15% to 36%, with an average of 25.5%. There was variation in how AF was defined and detected (Table S3). The most common definition, used by six studies, was utilizing the contemporaneous STS definition of postoperative AF, namely AF/flutter requiring treatment. The rate of off‐pump CABG ranged from 0% to 94.3% in the 13 studies that reported this data. Only four studies measured recurrence of AF.^8^, ^9^, ^10^, ^11^ Among patients with NOAF, AF recurred in 22% to 39% of patients. Among patients without NOAF, AF occurred in 0% to 13% of patients. Three studies reported the rate of anticoagulation.^12^, ^13^, ^14^ In those studies, patients who developed NOAF were anticoagulated 12% to 21% of the time. Patients who did not develop NOAF were anticoagulated 1% to 6% of the time.

### 3.3 Impact of NOAF on stroke and mortality

**FIGURE 2:**
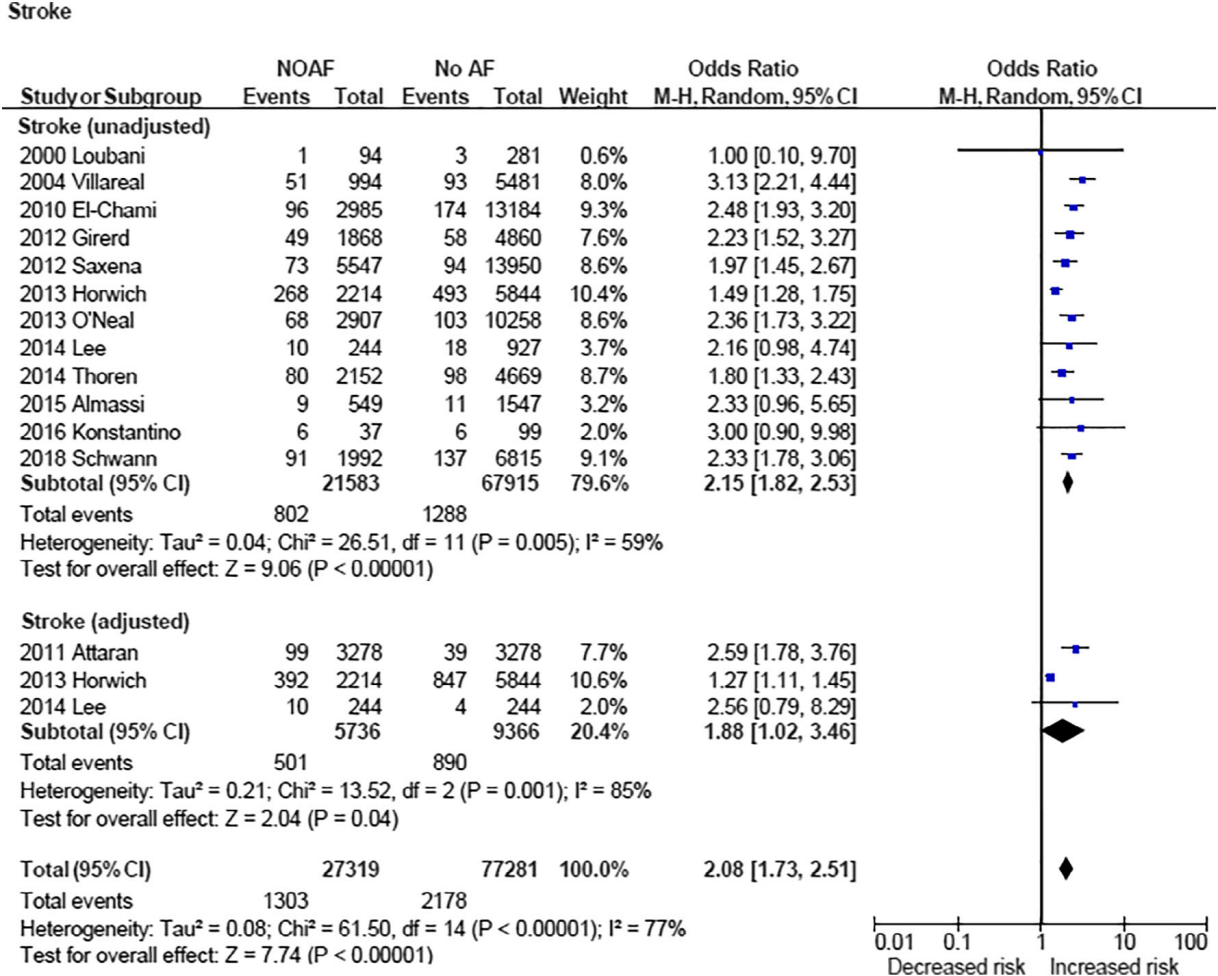
Forest plots (unadjusted and adjusted)—overall increased risk of stroke associated with new‐onset atrial fibrillation. CI, confidence interval; M‐H, Mantel‐Haenszel; NOAF, new‐onset atrial fibrillation

**FIGURE 3:**
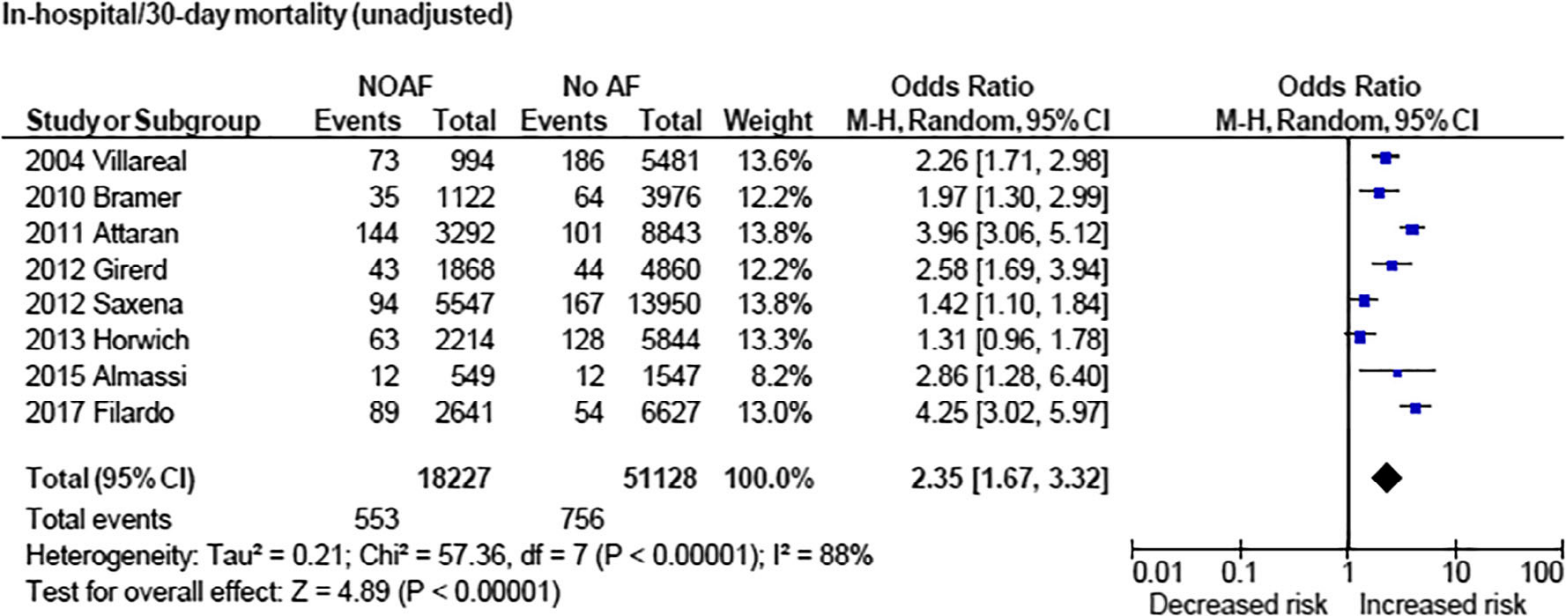
Forest plot (unadjusted)—overall increased risk of in‐hospital/30‐day mortality associated with new‐onset atrial fibrillation. CI, confidence interval; M‐H, Mantel‐Haenszel; NOAF, new‐onset atrial fibrillation

**FIGURE 4:**
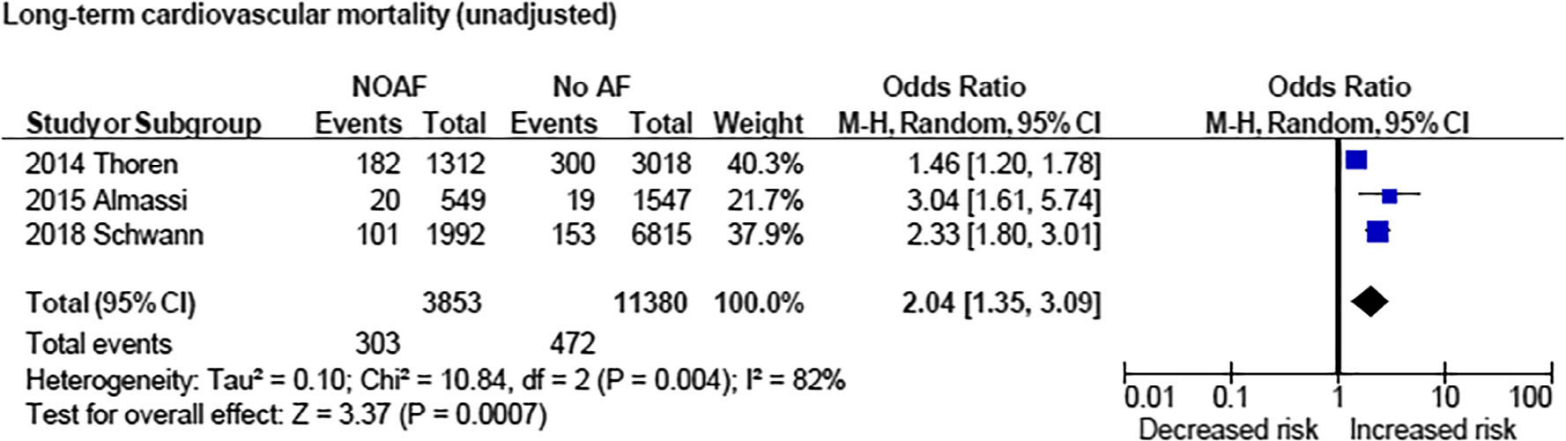
Forest plot (unadjusted)—overall increased risk of long‐term cardiovascular mortality associated with new‐onset atrial fibrillation. CI, confidence interval; M‐H, Mantel‐Haenszel; NOAF, new‐onset atrial fibrillation

**FIGURE 5:**
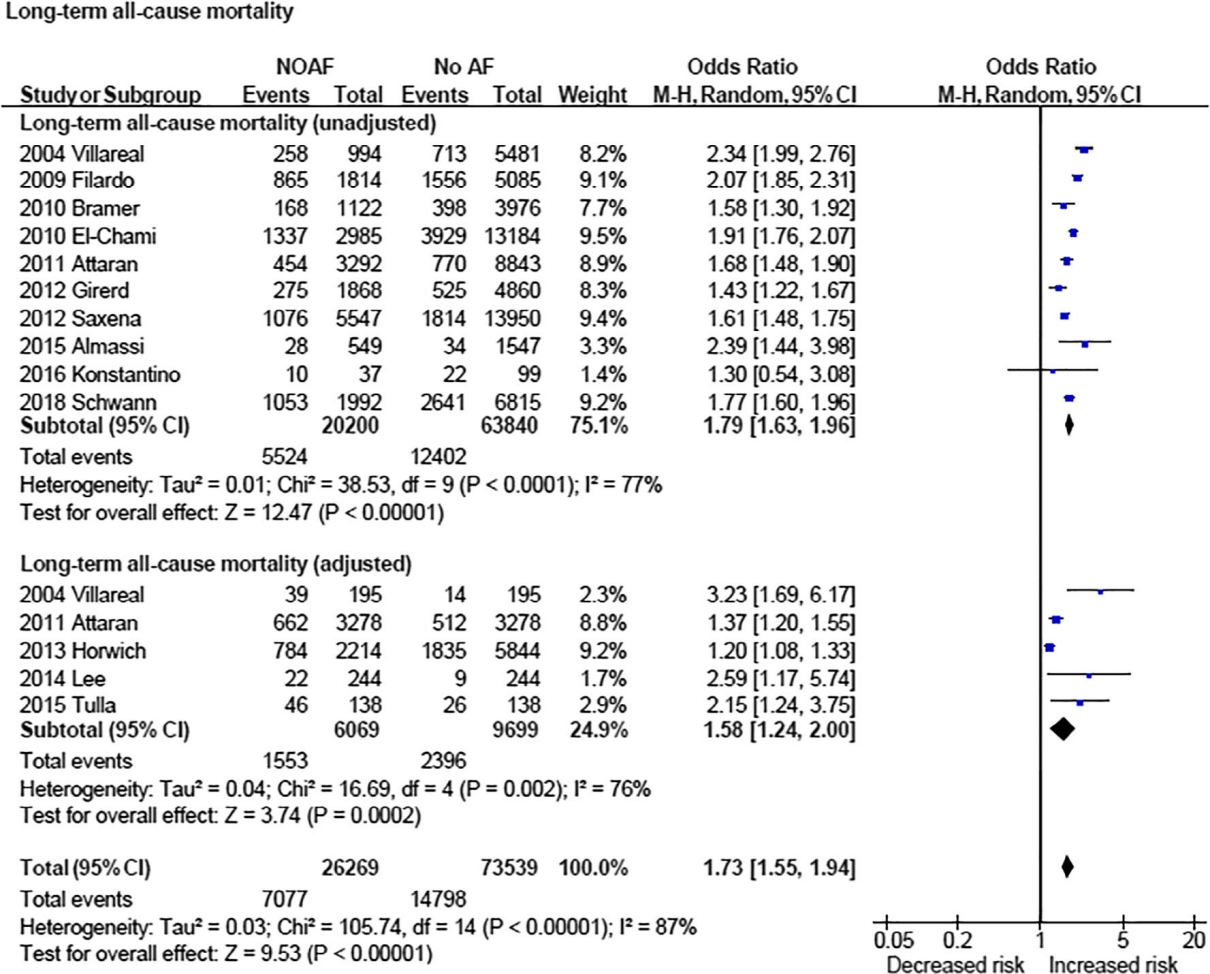
Forest plot (unadjusted and adjusted)—overall increased risk of long‐term all‐cause mortality associated with new‐onset atrial fibrillation. CI, confidence interval; M‐H, Mantel‐Haenszel; NOAF, new‐onset atrial fibrillation

NOAF was associated with increased risk of stroke when examining unadjusted data (OR 2.15 [1.82, 2.53] [*P* < .00001]) as well as adjusted data (OR 1.88 [1.02, 3.46] [*P* = .04]; Figure 2). NOAF was associated with increased 30‐day/hospital mortality (OR 2.35 [1.67, 3.32] [*P* < .00001]; Figure 3) and long‐term cardiovascular mortality (OR 2.04 [1.35, 3.09] [*P* = .0007]; Figure 4). NOAF was associated with increased long‐term all‐cause mortality (unadjusted OR 1.79 [1.63, 1.96] [*P* < 0.00001]; adjusted OR 1.58 [1.24, 2.00] [*P* = .0002]; Figure 5). We conducted subgroup analyses stratified by study quality, which yielded similar results. We defined high‐quality studies as scoring 8 or 9 on the Newcastle‐Ottawa scale. NOAF remained associated with increased long‐term all‐cause mortality when high‐quality studies were analyzed alone (unadjusted OR 1.79 [1.65, 1.94] [*P* < .00001]; adjusted OR 1.74 [1.16, 2.62] [*P* = .008]).

### 3.4 Quality Assessment

There was substantial heterogeneity among studies. Among studies reporting stroke outcomes, there was substantial heterogeneity in the unadjusted data (*I* ^2^ = 59%) and adjusted data (*I* ^2^ = 85%). The data on 30‐day/hospital mortality had substantial heterogeneity (*I* ^2^ = 88%), as did the data on long‐term cardiovascular mortality (*I* ^2^ = 82%). Among the studies that reported long‐term all‐cause mortality outcomes, the degree of heterogeneity was substantial both for unadjusted data (*I* ^2^ = 77%) and adjusted data (*I* ^2^ = 76%). The degree of heterogeneity decreased when high‐quality studies were analyzed alone. For example, the degree of heterogeneity for long‐term mortality was lower for both unadjusted outcomes (*I* ^2^ = 69%) and adjusted outcomes (*I* ^2^ = 57%).

We assessed study quality using the Newcastle‐Ottawa Scale. Ninety percent of the studies scored 7 or higher (out of 9). There were two common issues we identified. Several of the studies used a multivariable analysis but did not explicitly state the variables included in that analysis. In addition, 75% of studies did not report, or did not have access to, data on follow‐up rates (Table S1).

## 4 DISCUSSION

Our main finding is that the development of NOAF after isolated CABG is associated with increased risk of stroke as well as short‐term, long‐term, and cardiovascular mortality. The unique aspect of this meta‐analysis is the focus on NOAF outcomes in patients undergoing CABG alone, unlike prior meta‐analyses that have reported on “all‐comers” cardiac surgery.^3^, ^4^, ^5^ We excluded patients who underwent concomitant valvular procedures for two reasons. First, we considered that valvular procedures may predispose to a scarred atrial substrate, which could independently predispose to development of atrial arrhythmias. Second, patients undergoing valvular procedures may be anticoagulated a higher rate, reducing their risk of stroke. While the effect size appears to be larger in our meta‐analysis, it is primarily owing to the population of interest. Other meta‐analyses derive from patients who undergo valvular surgeries. We speculate a higher baseline rate of anticoagulation could skew to a lower effect size in this population.^5^

We were able to use adjusted data for the outcomes of stroke and long‐term all‐cause mortality. The increased risk associated with NOAF remained significant in the adjusted data. There are several potential confounding factors in this population, including surgical technique, comorbidities, and demographic characteristics. The increased risk of poor outcomes seen even in the adjusted data lends further support to the argument that NOAF independently may portend poor outcomes.

There are several limitations to this analysis. Although there are several studies that have analyzed impact of AF in the postoperative setting, only 19 studies met our inclusion criteria for two reasons. The majority of these studies were retrospective. There was substantial heterogeneity between studies. This was not entirely unexpected, given the differences in populations included in these studies. However, there was variation among studies in how AF was defined and detected, which has been shown to have mortality implications. Among patients who develop NOAF after CABG, having classic NOAF (by STS definition) was associated with reduced mortality. Those patients who had NOAF that was missed by the STS definition had significantly higher risk‐adjusted 30‐day mortality.^15^

We were unable to conduct systematic AF recurrence during follow‐up period owing to lack of studies reporting this consistently. Of the studies available, the recurrence rates were as high as 39%.^8^, ^9^, ^10^, ^11^ Unfortunately, none of the studies stratified outcomes based on recurrence of AF.

The timing of stroke varied between studies. Short‐term stroke rates were reported in three studies and showed increased stroke rate in the group with NOAF.^12^, ^16^, ^17^ The majority of studies reported stroke at greater than 1 year of follow‐up. There were equivocal results for long‐term stroke rates, with rates in the group with NOAF ranging from similar to the group without AF^8^, ^9^, ^11^, ^18^ to higher than the group without AF.^13^, ^14^, ^19^, ^20^, ^21^, ^22^

None of the studies explicitly excluded patients with prior stroke. In five studies, there was a significantly greater history of stroke in the NOAF group compared to the group without AF.^12^, ^13^, ^16^, ^19^, ^23^ In six of the other studies, either there was no significant difference in the history of stroke in the NOAF group compared to the group without AF,^10^, ^15^ or adjusted outcomes or matched groups were used.^14^, ^17^, ^21^, ^22^ It is likely that the unadjusted outcomes were affected by prior history of stroke. However, the association of NOAF with stroke and mortality was found even in the meta‐analysis of adjusted outcomes, indicating that NOAF is likely an independent risk factor.

The use of off‐pump CABG varied widely among these studies. The impact of off‐pump CABG on NOAF is unclear. Saxena et al showed that those patients who developed AF were significantly less likely to have undergone an off‐pump procedure.^16^ Girerd et al showed no difference.^17^ Schwann et al. showed that patients who developed NOAF were shown to have longer cardiopulmonary bypass and aortic cross‐clamp times than those without NOAF, even when receiving similar numbers of bypass grafts.^21^ None of the studies reported on the prevalence of left atrial appendage occlusion. Left atrial appendage occlusion has been associated with decreased risk of thromboembolism among patients discharged without anticoagulation.^24^ However, it has also been associated with increased risk of complications, including NOAF.^25^, ^26^ The unreported use of left atrial appendage occlusion could have impacted our results.

There are several mechanisms by which AF may lead to adverse outcomes. AF causes a number of physiologic abnormalities: left atrial stasis leading to thromboembolism, loss of atrioventricular synchrony, and decreased cerebral perfusion due to irregular ventricular contractions.^21^ There is a theoretical mechanism by which irregular ventricular contractions, leading to decreased cerebral circulation, may predispose patients to noncardioembolic stroke.^22^, ^27^ NOAF may a surrogate for general poor health, or for a generalized inflammatory state that leads to increased risk of cardiovascular events.^17^ A point supporting this theory is that stroke accounts for less than 10% of deaths in patients developing AF.^21^

Whether anticoagulation would improve outcomes in this patient population is not clear. In one study, those patients discharged on warfarin had a 22% relative reduction in mortality over a mean follow‐up of 6 years, compared to those discharged without warfarin.^7^ This study included over 600 patients with NOAF discharged on warfarin. One of the other studies included in this meta‐analysis did not find a mortality benefit, but was smaller, at 139 patients.^12^ There was wide variability in the practice patterns, with anticoagulation rates ranging from 12% to 21% in NOAF patients to 1% to 6% in the non‐NOAF category.^12^, ^13^, ^14^ The existing guidelines suggest that it is reasonable to manage NOAF with anticoagulation and cardioversion if AF does not spontaneously revert to AF during follow‐up, but no recommendation is made for those patients who develop NOAF and then revert to sinus rhythm.^28^ The question of rate vs rhythm control, however, has been previously studied. In patients with NOAF after cardiac surgery, rate and rhythm control have been shown be associated with similar rates of persistent AF and similar rates of complications.^29^

These results suggest two avenues for further research. The first is elucidating the true incidence of long‐term AF in this patient population. Implantable cardiac monitors provide a means with which to study this topic. Determining which patients have long‐term AF will further delineate which outcomes may due to AF and which outcomes are due to comorbidities. The second avenue of further research would guide the role of anticoagulation: whether it should be prescribed to all patients with NOAF, only those who have recurrent NOAF, or only those with concomitant indications. There is a current multicenter randomized trial comparing oral anticoagulation to no oral anticoagulation in patients who develop NOAF after CABG, which may provide further information on this topic.^30^

## 5 CONCLUSIONS

NOAF is a common outcome following isolated CABG. It is associated with an increased risk of stroke and long‐term mortality, even when adjusted for confounding variables. It is also associated with an increased risk of short‐term mortality, an outcome for which we only had unadjusted data. Based on the existing data, NOAF is independently associated with worse outcomes. Early recognition of NOAF may help risk‐stratify patients for closer follow‐up and monitoring. The outcomes analyzed here raise the question of whether anticoagulation would improve outcomes in these patients. Further research, including randomized controlled trials, is needed to answer this question.

## Supporting information


**Table S1** Newcastle‐Ottawa Scale quality assessment results
**Table S2.** Search strategy
**Table S3.** Study characteristics. NOAF indicates new‐onset atrial fibrillation; SVA, supraventricular arrhythmia; AFl, atrial flutter; NR, not reported; LOS, length of stay; MI, myocardial infarction.
