# Supplementary material for: New‐onset atrial fibrillation and outcomes following isolated coronary artery bypass surgery: A systematic review and meta‐analysis
Source: Clin Cardiol. 2020 Jul 21;43(9):928–34. doi: 10.1002/clc.23414 (PMC7462196; doi:10.1002/clc.23414)
Supplement: Supplementary file 1 — Table S1 Newcastle‐Ottawa Scale quality assessment results Table S2. Search strategy Table S3. Study characteristics. NOAF indicates new‐onset atrial fibrillation; SVA, supraventricular arrhythmia; AFl, atrial flutter; NR, not reported; LOS, length of stay; MI, myocardial infarction. [file CLC-43-928-s001.docx]

**Table S1**

Newcastle-Ottawa Quality Assessment Results

|  | Selection  (out of 4) | Comparability (out of 2) | Outcome  (out of 3) |
| --- | --- | --- | --- |
| Loubani | 4 | 2 | 2 |
| Villareal | 4 | 0 | 3 |
| Filardo 2009 | 4 | 2 | 3 |
| El-Chami | 4 | 2 | 3 |
| Bramer | 4 | 2 | 2 |
| Attaran | 4 | 2 | 2 |
| Saxena | 4 | 2 | 2 |
| Girerd | 4 | 0 | 3 |
| Horwich | 4 | 1 | 2 |
| Almassi | 4 | 2 | 2 |
| O'Neal | 4 | 2 | 2 |
| Thoren | 4 | 2 | 2 |
| Lee | 4 | 2 | 2 |
| Tulla | 4 | 2 | 2 |
| Konstantino | 4 | 0 | 3 |
| Schwann | 4 | 2 | 2 |
| Filardo 2017 | 4 | 1 | 2 |
| Al-Shaar | 4 | 2 | 2 |
| Sahin | 4 | 0 | 2 |

**Table S2.** Search strategy

PubMed/MEDLINE:

(secondary OR new onset OR recent onset) AND (atrial fibrillation OR atrial flutter OR arrhythmia OR atrial fibrillation[MeSH Terms] OR atrial flutter[MeSH Terms] OR arrhythmia[MeSH Terms]) AND (cardiac surgery OR bypass surgery OR post operative OR cardiac surgery[MeSH Terms] OR coronary artery bypass surgery[MeSH Terms] OR period, postoperative[MeSH Terms] OR revascularization OR myocardial revascularization[MeSH Terms] OR revascular*)

CENTRAL:

{(secondary OR new onset OR recent onset) AND (atrial fibrillation OR atrial flutter OR arrhythmia OR atrial fibrillation[MeSH descriptor; explode all trees] OR atrial flutter[MeSH descriptor; explode all trees] OR arrhythmias, cardiac [MeSH descriptor; explode all trees]) AND (cardiac surgery OR bypass surgery OR post op* OR revascularization OR coronary artery bypass[MeSH descriptor; explode all trees] OR postoperative complication[MeSH descriptor; explode all trees] OR myocardial revascularization[MeSH descriptor; explode all trees]} NOT (“accession number” near pubmed)

Web of Science

TS=(secondary OR new onset OR recent onset) AND TS=(atrial fibrillation OR atrial flutter OR arrhythmia) AND TS=(cardiac surgery OR bypass surgery OR post operative OR revascularization)

**Table S3. Study characteristics**

| Author | Study type | Multi-center vs single center | New-castle Ottawa scale | Total pts | NOAF pts | AF definition | Anti-coagulation on d/c | % off-pump | % Follow-up | Outcomes |
| --- | --- | --- | --- | --- | --- | --- | --- | --- | --- | --- |
| Loubani (2000)^18^ | Retrospective cohort | Single center | 8 | 375 | 94 | SVA, including AF (89%), Afl, SVT | NR^†^ | 0 | NR | Hosp LOS, stroke. Note, there was no hospital mortality. |
| Villareal RP (2004)^14^ | Retrospective cohort | Single center | 7 | 6475 | 994 | Documentation of AF of any duration at any time on physician assessment of an EKG or rhythm strip | 14% (AF group), 6.25% (no AF group) | NR | 100 | Early mortality (<=30d), MI, stroke, hosp. LOS, 1-yr mortality, 4-yr mortality |
| Filardo G (2009)^19^ | Retrospective cohort | Single center | 9 | 6899 | 1814 | AF/Afl requiring treatment (STS definition) | NR | 8.9-9.2 | 100 | 5-yr and 10-yr survival |
| El-Chami MF (2010)^20^ | Retrospective cohort | Multicenter (2) | 9 | 16169 | 2985 | AF/Afl requiring treatment (STS definition) | 20.5% (POAF), 4.1% (no AF) | NR | 100 (for survival data) | 1-yr and 10-yr mortality |
| Bramer S (2010)^21^ | Retrospective cohort | Single center | 8 | 5098 | 1122 | Any evidence of AF by EKG or tele, lasting at least 30 min. | If AF lasted >48h | 11 | 99.4 | Early mortality (hosp. or 30d), late mortality, 5-yr mortality |
| Attaran S (2011)^12^ | Retrospective cohort | Single center | 8 | 12135 | 3292 | AF confirmed on EKG for any length of time | NR | NR | NR | 30-d mortality, 5-yr mortality, 10-yr mortality |
| Saxena A (2012)^9^ | Prospective cohort | Multicenter (20) | 8 | 19497 | 5547 | New AF, diagnosed by EKG or tele, that required treatment | NR | 5.7-8.8 | NR | 30-d mortality, stroke, late mortality (1,3,5, and 7-yr), LOS, ICU LOS |
| Girerd N (2012)^10^ | Retrospective cohort | Single center | 7 | 6728 | 1868 | Any sustained episode requiring treatment | NR | 4.7 | 80.4 (for long-term data) | Early mortality (hosp or 30-d), late mortality (6-yr), stroke |
| Horwich P (2013)^22^ | Retrospective cohort | Single center | 7 | 8058 | 2214 | AF/Afl requiring treatment (STS definition) | 11.9% (POAF), 1.2% (no AF) | 4.9 | NR | Early mortality (in-hospital), late mortality (in-hospital, 1, 5, and 10-yr), stroke (1, 5, and 10-yr) |
| O'Neal WT (2013)^23^ | Retrospective cohort | Single center | 8 | 13165 | 2907 | AF lasting longer than 1 hour and requiring treatment according to STS criteria | Yes, if in AF | NR | NR | Stroke |
| Thoren E (2014)^24^ | Prospective cohort | Single center | 8 | 6821 | 2152 | AF lasting more than 30s on EKG or telemetry | NR | 4 | NR | Late mortality (>30d, 10-yr), cardiac mortality, stroke |
| Lee SH (2014)^25^ | Retrospective cohort | Single center | 8 | 1171 | 244 | New AF seen on EKG or telemetry during the first 10 days after surgery | Yes, if in AF and no contraindications | 94.3 | 93.3 (for >1yr follow up) | Mortality and stroke (both at 10d to 3mo, 3-12 mo, and >1yr), hosp. LOS |
| Almassi GH (2015)^26^ | Retrospective cohort | Multicenter (18) | 8 | 2096 | 549 | Any abnormal atrial-originated rhythm without discernible P waves on electrocardiogram lasting more than 30 minutes | NR | 49.8 | NR | Stroke, mortality (30-d, 1-yr), cardiac mortality (30-d, 1-yr) |
| Tulla H (2015)^8^ | Retrospective cohort | Single center | 8 | 276 | 138 | AF seen on EKG lasting more than 5 min | All AF patients | 5.8 | NR | Hosp. LOS, ICU LOS, TIA or stroke |
| Konstantino (2016)^27^ | Retrospective cohort | Single center | 7 | 136 | 37 | ICD-9 codes, either upon discharge or at outpatient follow-up | NR (65% during follow-up period) | NR | NR | Mortality and CVA (included Kaplan-Meier curves up to 10 years) |
| Schwann TA (2018)^11^ | Retrospective cohort | Multicenter (3) | 8 | 8807 | 1992 | AF/Afl requiring treatment (STS definition) |  |  |  | Mortality (1, 3, 6, 9, 12, and 15-yr) |
| Filardo G (2017)^7^ | Retrospective cohort | Multicenter (5) | 7 | 9268 | 2641 | AF/Afl requiring treatment (STS definition) | NR | 38 | NR | Mortality (30-d) |
| Al-Shaar L (2014)^13^ | Retrospective cohort | Single center | 8 | 6305 | 1211 | AF/Afl requiring treatment | Persistent/ recurrent POAF | 4 | NR | Mortality (2, 4, 8, 12, 16 yrs) |
| Sahin, M (2016)^28^ | Retrospective cohort | Single center | 6 | 149 | 55 | NR | NR | 0 | NR | Hosp LOS, ICU LOS |

^†^NR = not reported
